# Separation of Levofloxacin from Industry Effluents Using Novel Magnetic Nanocomposite and Membranes Hybrid Processes

**DOI:** 10.1155/2019/5276841

**Published:** 2019-04-04

**Authors:** Azmat Ullah, Muhammad Zahoor, Sultan Alam, Riaz Ullah, Ali S. Alqahtani, Hafiz Majid Mahmood

**Affiliations:** ^1^Department of Chemistry, University of Malakand, Chakdara, Dir Lower, 18800 KPK, Pakistan; ^2^Medicinal, Aromatic and Poisonous Plants Research Center (MAPRC), College of Pharmacy, King Saud University, P.O. Box 2457, Riyadh 11451, Saudi Arabia; ^3^Department of Pharmacognosy, College of Pharmacy, King Saud University, P.O. Box 2457, Riyadh 11451, Saudi Arabia; ^4^Department of Pharmacology, College of Pharmacy, King Saud University, P.O. Box 2457, Riyadh 11451, Saudi Arabia

## Abstract

Magnetic carbon nanocomposite (MCN) was synthesized from waste biomass precursor, pineapple. The prepared adsorbent was characterized using different instrumental techniques and was used to remove levofloxacin (LEV) from effluents. The maximum sorption of LEV was observed at pH 7. Pseudo-2nd-order (PSO) kinetic was found to be the best model that fits well the adsorption kinetics data. For Langmuir adsorption isotherm, the R^2^ value was higher as compared with other isotherms. The Van't Hoff equation was used for thermodynamic parameters determinations. ΔS° (standard entropy) was positive and ΔG° (standard Gibb's free energy) was negative: -0.37, -1.81, and -3.73 kJmol^−1^ corresponding to 25, 40, and 60°C. The negative values of ΔG° at different temperatures stipulate that the adsorption of LEV was spontaneous in nature and adsorbent has a considerable affinity for LEV molecules. The MCN was then utilized in hybrid way by connecting with ultrafiltration (UF), nanofiltration (NF), and reverse osmosis (RO) membranes in series and as a result enhanced permeate fluxes were observed. The percent retention of LEV molecules was lower with UF membrane and with NF it was 96%, while it was 100% with RO. For MCN/UF and MCN/NF systems, improvement in % retention was recorded.

## 1. Introduction

Levofloxacin (LEV), a fluoroquinolones (FQs), is the synthetic antibiotics used to treat infectious diseases. FQs are safe, broad spectrum drugs, having high tolerance limit and good potency [1–3]. However, these drugs are poorly absorbed and metabolized in humans/animals bodies and are largely excreted through human/animals urine and feces in their active pharmacological forms [4, 5]. According to previous reports, FQs are the most repeatedly detected antibiotics throughout the world followed by other classes of antibiotics such as tetracycline, sulfonamides, and macrolides in the aqueous environment [6, 7]. The continuous entry of FQs even at low concentration in aqueous media is a threat towards the safety of drinking/fresh water and aquatic ecology.

The already utilized methods for the remediation of antibiotics from waters include membrane separation processes [8, 9], ozonation [10], advanced oxidation processes [11], photochemical degradation [12], and adsorption methods [13]. Amongst them, adsorption is preferred due to low cost, no by-product formation, and easy handling. Different sorbent materials are used for the removal of FQs such as kaolinite [14], magnetite [15], and carbon nanomaterials [16]. The supremacy of adsorption over other methods is due to the usage of carbonaceous materials [17]. However, there is difficulty of recovery and settling very slowly in slurry after use [18–21]. To resolve this issue, activated carbon is presently renewed into magnetic carbon nanocomposites (MCN) which are far better than activated charcoal (carbon) due to its magnetic properties; additionally MCN also has an excellent sorption capacity [21, 22].

Membrane methods like UF, NF, and RO are the developing technologies utilized globally for the decontamination of portable and industrial water. Though synthetic organic matter (SOM) has a serious impact on the efficiency of these membranes, such ingredients get sorbed on the exterior of membranes and block the tiny pores of membranes. Activated charcoal has been utilized prior to membrane to avoid their adsorption on membrane surfaces. Initially it was presumed that if the loaded activated carbon enter membrane, network will produce a permeable layer over the surface of membrane and will not affect its permeate flux. Yet, later on, it was verified that permeable cake also has an effect on the membrane efficiencies. To resolve this problem, some authors used magnetic activated carbon in combination with membrane which can then be efficiently removed before entering membranes through application of external magnetic processes [18–21].

The goal of this study was to prepare MCN from biomass precursor of pineapples and characterize and use it for the elimination of LEV from the aqueous solutions by batch adsorption and adsorption/membrane hybrid method.

## 2. Experimental

### 2.1. Materials

Analytical grade chemicals were used in all experiments. Levofloxacin hemihydrate was collected from local pharma in Swat. The characteristic features of LEV are tabulated in Table 1.

### 2.2. Synthesis and Characterization of MCN

Waste biomass precursors of pineapple were used to produce the novel adsorbent. The dried biomass was transferred into a container containing a suspension of ferric chloride and ferrous sulphate in 2:1. Concentrated NaOH solution was added to the mixture at 70-80°C with continuous stirring. The mixture was then charred and ignited. After ignition, the product was washed away with HCl solution and distilled water a number of times to achieve the neutral pH. The final product was dried at 70°C in an electric oven for 24 hours. The prepared sample MCN was characterized by EDX, SEM, XRD, FTIR, TG/DTA, surface area analyzer, and pH_(pzc)_.

### 2.3. Optimization Experiments

To find out optimum condition for best removal of LEV, 50 mL of LEV solutions was stirred with varying dosage of MCN at 298K for a specific period of time. Maximum removal of LEV was achieved with a dose of 0.04 g and was therefore used in the subsequent experiments. The effect of pH was evaluated and optimum pH was found to be 7. Containers used in the study were checked for the adsorption of LEV in controlled experiments without adding adsorbent. The adsorption of LEV by the container walls was negligible under the given conditions. The concentration of the LEV in aqueous solution was determined using UV/visible spectrophotometer at 280 nm.

The sorption tests of LEV were studied in the aqueous solutions. First of all, the stock solution was synthesized by dissolving the suitable amounts of LEV in double distilled water. The general procedure utilized in this study was to permit a definite quantity of MCN in 200 mL glass reagent bottle having definite volume in mL of LEV solution of specified concentration in mgL^−1^, according to the prerequisite of analysis. The glass reagent bottles were set in a rotary shaker and were oscillated at a speed of 150 revolution per minute (rpm) for definite duration of time at room temperature (298K). Dilute acid (HCl) and base (NaOH) solutions were used to adjust the solution pH (0.1 molL^−1^). The MCN was detached from slurry through application of a magnetic bar. Finally, the LEV solution in the glass reagent bottles was filtered. The filtrates were examined for the ratio of LEV in solution using UV/Visible spectrophotometer at 280 nm. The adsorbed LEV, q_e_ (mgg^−1^), was estimated utilizing the following formula:

(1) $$\begin{array}{c} q_{e}=\left(\;C_{o}-C_{e}\;\right)\frac{V}{W} \end{array}$$

In (1), C_o_ is initial concentration of LEV in mgL^−1^, C_e_ is equilibrium LEV concentration (mgL^−1^), q_e_ is amount of LEV sorbed on MCN (mgg^−1^), V is solution volume (L), and W is MCN mass (g).

The % R (percent removal) was determined as

(2) $$\begin{array}{c} \%R=\left(\;\frac{C_{o}-C_{e}}{C_{o}}\;\right)100 \end{array}$$

### 2.4. Membrane Processes and Adsorption/Membrane Hybrid Processes

**Figure 1:**
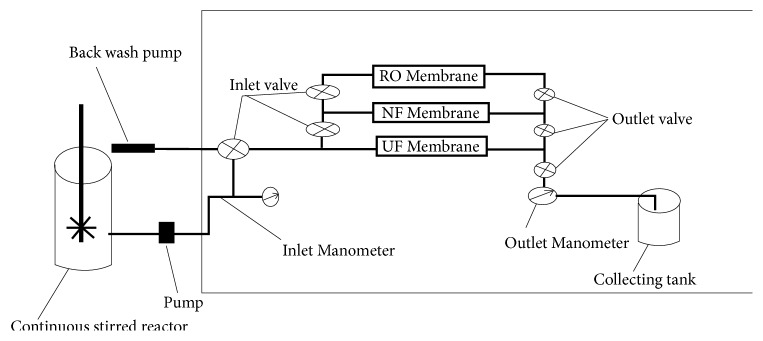
MCN/UF/NF/RO pilot plant.

For the determination of % retention of LEV and their impact on effluent fluxes (J) through three selected membranes, ultra-, nano-, and reverse osmosis membranes were utilized in this study. The characteristics properties of these membranes are tabulated in Table 2. The hybrid plant used in this study is shown in Figure 1. The selected membranes were washed with distilled water as instructed by the producer many times to equilibrate them. A 40mgL^−1^ LEV aqueous solution was utilized during all experimental cycles at 298K and 1.0 bar pressure. The % R of LEV and the decline in the flow rate by of each membrane without the addition of MCN were determined.

The pore blockage on membranes surfaces resulted in reduction of permeate fluxes when passing through naked membrane which was compensated through MCN adsorption in continuously stirred reactor. A specifically constructed plant was utilized for this purpose (Figure 1). The decrease in permeate flow due to intrinsic membrane resistance to water was noted. The LEV solutions were taken in 12 L vessel and allowed to pass through UF/NF/RO membranes with the help of a multispeed water pump. The % retention of LEV and their effect on permeate flow were noted. Now, continuously stirred reactors were connected with membrane system. Reactor MCN was added in a single dose. Before letting the effluents into membrane systems the MCN was separated from the slurry through application of external magnetic field.

Membrane parameters like % R of LEV and their influence on permeate flow were calculated using the following relation:

(3) $$\begin{array}{c} \%R=100\left(\;1-\frac{C_{p}}{C_{b}}\;\right) \end{array}$$

where C_b_ is the LEV concentration in bulk while C_p_ is permeate.

Permeate flux (J) Lm^−2^ h^−1^ of membranes were calculated utilizing the following relation:

(4) $$\begin{array}{c} J=\frac{1}{A}\frac{dV}{dt} \end{array}$$

where V is effluent volume and A is area of membrane.

After each successive experimental cycle, backwashing of each membrane was accomplished.

## 3. Results and Discussion

### 3.1. Characterization of Magnetic Carbon Nanocomposite Prepared from Biomass Precursors of Pineapple

**Figure 2:**
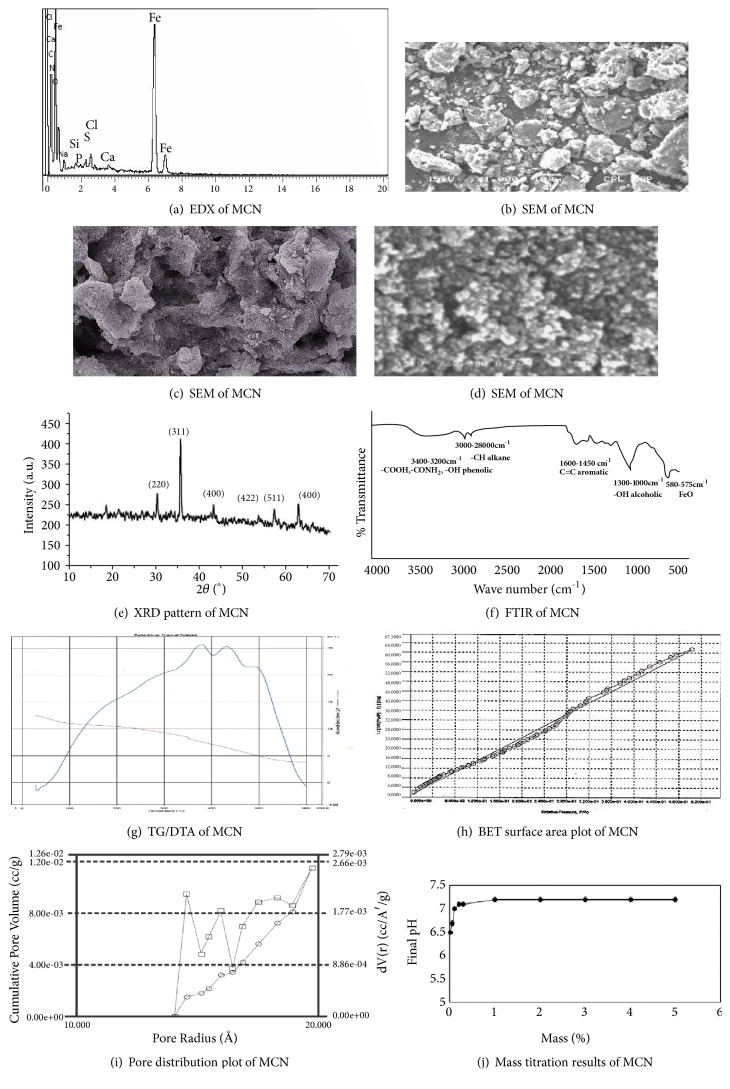
Characterization of MCN.

The magnetic behavior of prepared MCN was checked by subjecting it to a bar magnet and the strong attraction of MCN by a bar magnet indicated the formation of magnetic carbon nanocomposite. Electron dispersive X-ray analysis of pineapple MCN is represented in Figure 2(a). The figure shows the presence of elements such as Fe, O, and C, while calcium (Ca) and sodium (Na) were present as an impurity. Various peaks of iron and oxygen in MCN confirm the presence of magnetite [20].

SEM (scanning electron microscopy) of MCN with low and high magnification is given in Figures 2(b), 2(c), and 2(d). SEM images give information about the morphology of MCN. It was observed from SEM images that these composites have different shapes and sizes. The presence of white spots/patches in images shows the presence of water of crystallization of Fe_3_O_4_ in the MCN, while the SEM images also show the clump of particles due to contents of moisture sorbed in the sample. Moreover, SEM images explain the cubic crystalline structure of Fe_3_O_4_ [18–23].

Figure 2(e) displays the XRD pattern of MCN. A series of characteristics diffraction peaks of carbon coated with Fe_3_O_4_ magnetic nanoparticles was observed in the XRD pattern at 2*θ* of 30, 35.7, 44, 53, 57.95, and 62.5° corresponding to the diffraction indices of 220, 311, 400, 422, 511, and 400 of Fe_3_O_4_ clearly indicating the cubic crystalline structure magnetite [24]. Similar results of XRD patterns were previously reported by Zahoor et al. [20] and Mahdavi et al. [23]. Apart from the specific diffraction peaks of Fe_3_O_4_, small peaks of hematite were also observed.

Figure 2(f) shows the FTIR spectra of Fe_3_O_4_ coated carbon (MCN). The FTIR analysis indicated broad band absorption peaks at 3470-3200 cm^−1^ (related to surface functional groups such as phenol, -COOH, -CONH_2_, or physio sorbed H_2_O molecules on the surface of MCN), 3000-2800 cm^−1^ (related to –CH alkanes and), 1450-1600 cm^−1^ (related to C=C aromatic), 1300-1000 cm^−1^ (-OH alcoholic or R-O-R ), and 575-580 cm^−1^ (related to the presence of magnetite/maghemite Fe-O phase) [20].

The TGA/DTA curves of the MCN are shown in Figure 2(g), using initial mass of MCN 5.315 mg. The figure shows the 1st mass loss step (8%) at 20 to 100°C which was in fact loss of water of hydration/crystallization of Fe_3_O_4_/C. The 2nd mass loss step (26%) at 130 to 350°C and attributed to the thermal degradation of cellulosic materials present in MCN results in the formation of carbonaceous residues. From 400°C onward the MCN decomposes slowly up to 600°C and 3rd mass loss (47%) occurs at 400 to 540°C. From 400°C onward the MCN which decomposes slowly up to 600°C, a 3rd mass loss (47%) occurs which is considered to be phase transition from Fe_3_O_4_ to FeO, as the latter is thermodynamically stable above 570°C [23, 25]. Above 550°C no further loss in mass of MCN occurs. The final remains are a mixture of char and ash. Differential thermal analysis showed three endothermic peaks from 40°C to 450°C.

The surface area and pores distribution volume of the MCN are given in Figures 2(h) and 2(i), respectively. Different surface parameters were surface area = 39 m^2^g^−1^, total pore volume = 0.2 cm^3^g^–1^, and average pore diameter = 19.75A°.

The mass titration graph of MCN is given in Figure 2(j) from which the pH _(pzc)_ was calculated. Briefly different amounts of MCN (in the range of 0.01- 0.5 mass) were soaked in deionized water and sealed in test tubes (nitrogen atmosphere inside) and resulting pH values were measured after 24 hours. The point of zero charge of MCN was found to be 7.2 from the Figure 2(j).

### 3.2. Sorption Kinetics

**Figure 3:**
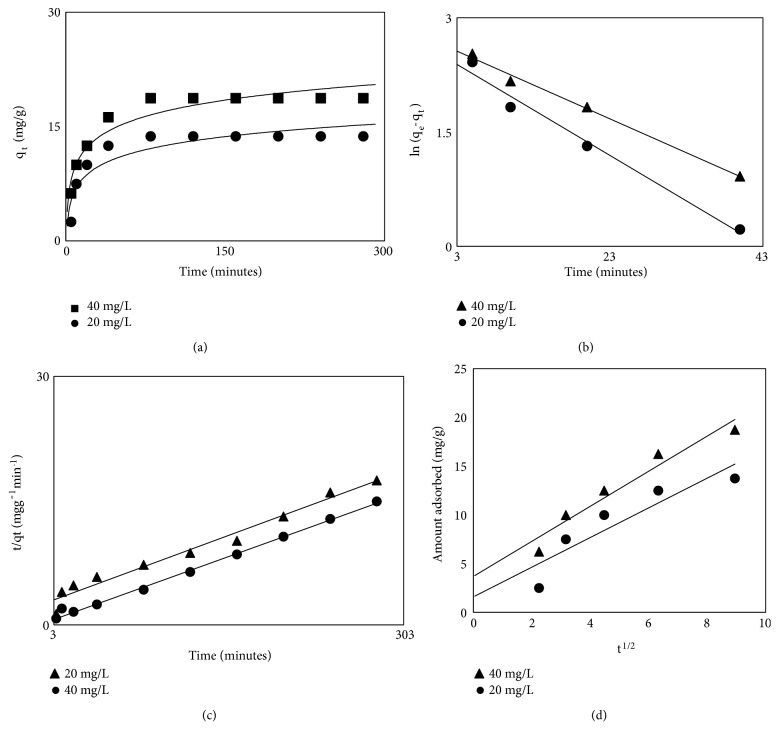
Adsorption kinetics plots of LEV onto pineapples magnetic carbon nanocomposite ((a)= effect of time, (b)= pseudo-1st-order, (c)= pseudo-2nd-order, and (d)= intraparticle diffusion).

The results of kinetic studies are shown in Figures 3(a), 3(b), 3(c), and 3(d). It was observed from the figure that the adsorption of LEV onto MCN was carried out at different contact time, i.e., from 5 to 280 minutes, using 20 and 40 mgL^−1^ initial LEV concentration. It is clear from Figure 3(a) that the adsorption of LEV was fast at the initial stage and equilibrium was attained in one-hour time. After establishment of equilibrium, no significant change was observed in the adsorption process. The fast adsorption of LEV at the initial stage was due to the availability of large number of vacant cites on prepared adsorbent. When these sites are occupied the rate of adsorption decreases gradually and finally reached equilibrium.

The pseudo-1st-order [26] and pseudo-2nd-order [27] kinetic models were applied to the kinetic of sorption data utilizing the following relations, respectively:

(5) ln⁡qe−qt=ln⁡qe−K1t

(6) tqt=1K2+tqe

where q_e_ = mgg^−1^ of LEV adsorbed at equilibrium whereas q_t_ is at any time interval and K_1_ = min^−1^ and K_2_ = gmg^−1^min^−1^ are the rate constant in the above equations. The pseudo-1st-order kinetic model is obtained by plotting ln (q_e_-q_t_) against t (Figure 3(b)), while 2nd-order model of kinetics is obtained by plotting t/q_t_ against t (Figure 3(c)). The values of rate constants K_1_ and K_2_ were calculated from the slopes and q_e_ from intercepts of linear plots (Table 3). The fit results of Figures 3(b) and 3(c) in terms of R^2^ values were compared and were found to be higher in the case of pseudo-second-order model than the 1st-order model (Table 3).

In order to determine the rate controlling step of sorption kinetics, the intraparticle diffusion model [28] of Weber and Morris was utilized and is given as follows:

(7) $$\begin{array}{c} q_{t}=K_{diff}t^{1/2}+C \end{array}$$

In (7), *q*_*t*_ is the amount of LEV sorbed in mgg^−1^ at any time t interval, K_diff_ is rate constant in mgg^−1^min^-1/2,^ and C is boundary layer thickness in mgg^−1^. The intraparticle diffusion plot is achieved by plotting. The constants K_diff_ and C are calculated from the slope and intercept of qt versus t^1/2^ plot, respectively. In order to know the kinetic mechanism of LEV adsorption from aqueous solution onto MCN prepared from waste biomass precursors of pineapple q_t_ was plotted verses t^1/2^ (Figure 3(d)). In figure there is an initial curve followed by a liner portion. The initial curve represents boundary film effect while linear portion presents intraparticle diffusion. The deviation of linear plots from the origin obviously indicates that LEV adsorption on the surface of MCN may have an additional rate controlling step [14–16]. The kinetic parameters K_diff_ and C of intraparticle diffusion model as well as R^2^ are listed in Table 3.

### 3.3. Adsorption Isotherm

**Figure 4:**
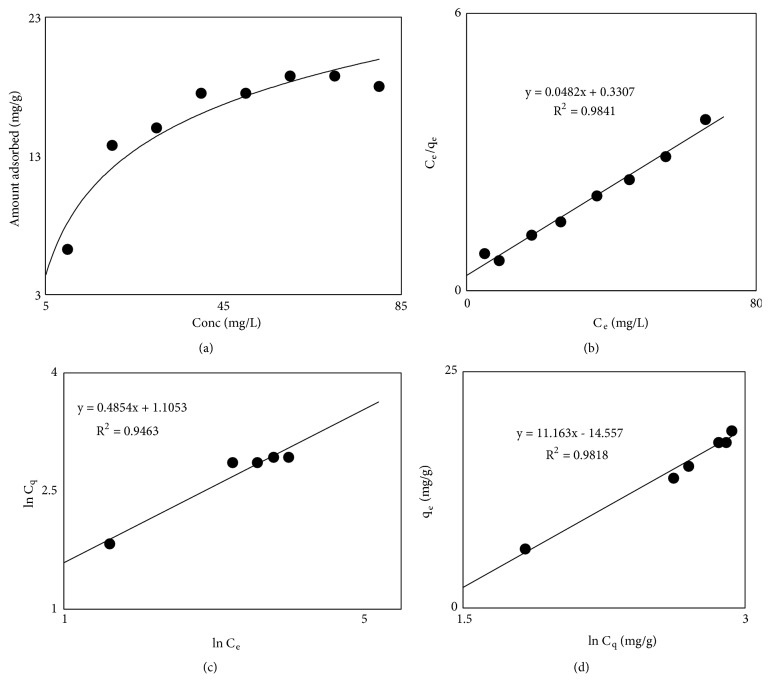
Adsorption isotherms of LEV onto pineapples magnetic carbon nanocomposite ((a)= effect of LEV concentration on adsorption, (b)= Langmuir isotherm, (c)= Freundlich isotherm, and (d)= Temkin isotherm).

To determine the effect of LEV concentration on adsorption different solutions of LEV were shaken with 0.04 g of adsorbent for 60 minutes. The extent of sorption was increased with the increase in concentration of the LEV which then reached to constant values (at higher concentrations, Figure 4(a)) as saturation of available active sites on the surface of MCN took place at high concentration.

For quantitative determination of the sorption of LEV on MCN, isotherm models like Langmuir, Freundlich, and Temkin were used.

The Langmuir isotherm in linear form can be given by the following relation:

(8) $$\begin{array}{c} \frac{C_{e}}{q_{e}}=\frac{1}{K_{L}q_{m}}+\frac{C_{e}}{q_{m}} \end{array}$$

In (8), C_e_ is equilibrium concentration of the LEV in mgL^−1^, qe is the amount LEV sorbed in mgg^−1^, q_m_ in mgg^−1^ is the maximum sorption capacity of LEV, and K_L_ in Lmg^−1^ are Langmuir constant related to adsorption energy, respectively. The Langmuir plot of specific adsorption is obtained by plotting C_e_/q_e_ versus C_e_ for the sorption of LEV shown in Figure 4(b). The constants K_L_ and q_m_ of Langmuir model were calculated from the intercept and slope of the plot, respectively, and are tabulated in Table 4. Low value of maximum sorption capacity (20.75 mgg^−1^) of MCN is because the micropores blockage of MCN is due to impregnation of Fe_3_O_4_ nanoparticles.

The Freundlich isotherm is an empirical equation employed to explain heterogeneous schemes. The linear form of the Freundlich model is given by the following equation:

(9) $$\begin{array}{c} \ln q_{e}=\ln K+\ln\frac{C_{e}}{n} \end{array}$$

In (9), q_e_ in mgg^−1^ is the amount of LEV adsorbed, C_e_ in mgL^−1^ is the equilibrium concentration of LEV, and n and k are Freundlich constants. The Freundlich plot is obtained by plotting ln C_e_ versus ln q_e_ and the values of K and 1/n were calculated from slope and intercept of the plot (Table 4). For LEV adsorption on adsorbent prepared from waste biomass precursors of pineapple, the Freundlich isotherm model is given in Figure 4(c). The value of 1/n < 1 in Freundlich model is indicative of normal adsorption process [29].

The Temkin isotherm in the linear form can be presented as follows:

(10) $$\begin{array}{c} C_{q}=\beta \ln\alpha +\beta \ln C_{e} \end{array}$$

In (10), C_q_ is the amount of LEV adsorbed in mgg^−1^ on the surface of MCN, C_e_ is the equilibrium concentration in mgL^−1^ of LEV and *β*=RT/b, R (8.314 Jmol^−1^k^−1^) is a general gas constant, T is absolute temperature in kelvin (K), and b is correlated to the heat of adsorption.  Figure 4(d) shows Temkin plot (C_q_ versus lnC_e_); the different constants (*β* and *β*ln*α*) of Temkin model are given in Table 4. Different values in Table 4 clearly indicated that Langmuir adsorption model of isotherm fitted well to the adsorption isotherm data with q_m_ (20.75 mgg^−1^) and R^2^ (0.984) compared to the other two isotherm models.

### 3.4. Impact of pH and MCN Dose on the Sorption of LEV

**Figure 5:**
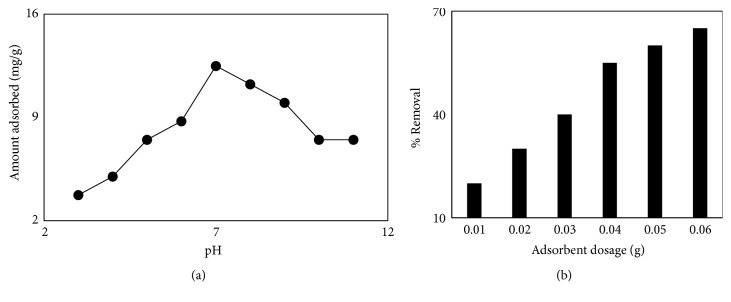
Effect of pH and adsorbent dosage on LEV adsorption.

To find out whether or not the pH affects the removal of LEV by MCN adsorption experiments were conducted in the pH range of 3-11.  Figure 5(a) shows that with an increase in pH from 3 to 7, the removal of LEV increases. In the range of pH 3-7 LEV molecules occurring in cationic form LEV^+^, as pH increases the cationic form of LEV^+^ gradually decreases and LEV molecules are converted to LEV^+-^ (zwitter ionic form). As the pH of LEV solution increases from 7 and becomes alkaline a gradual decrease in LEV removal takes place. At alkaline pH LEV molecules in solution exists in the anionic form (LEV^−^). The aspect for maximum exclusion of LEV molecules at pH < 7 or at pH = 7 may be owing to cationic interchange [24, 29–31].

The impact of dosage of MCN in the range of 0.01–0.06 g at 30 mgL^−1^ LEV concentration was investigated at pH 7 and 25°C.  Figure 5(b) shows the results of MCN dosage. The figure shows that LEV molecules removal rises speedily with rise in MCN dose, i.e., 0.01-0.04 g. The onwards rise is very sluggish. The preliminary fast rise in remediation of LEV may be due to larger number of sorption sites. So, 0.04 g of the MCN dosage was selected as optimum dosage and utilized in different sorption experiments.

### 3.5. Retention of Selected Antibiotic by Membranes and Adsorption/Membrane Hybrid Processes

**Figure 6:**
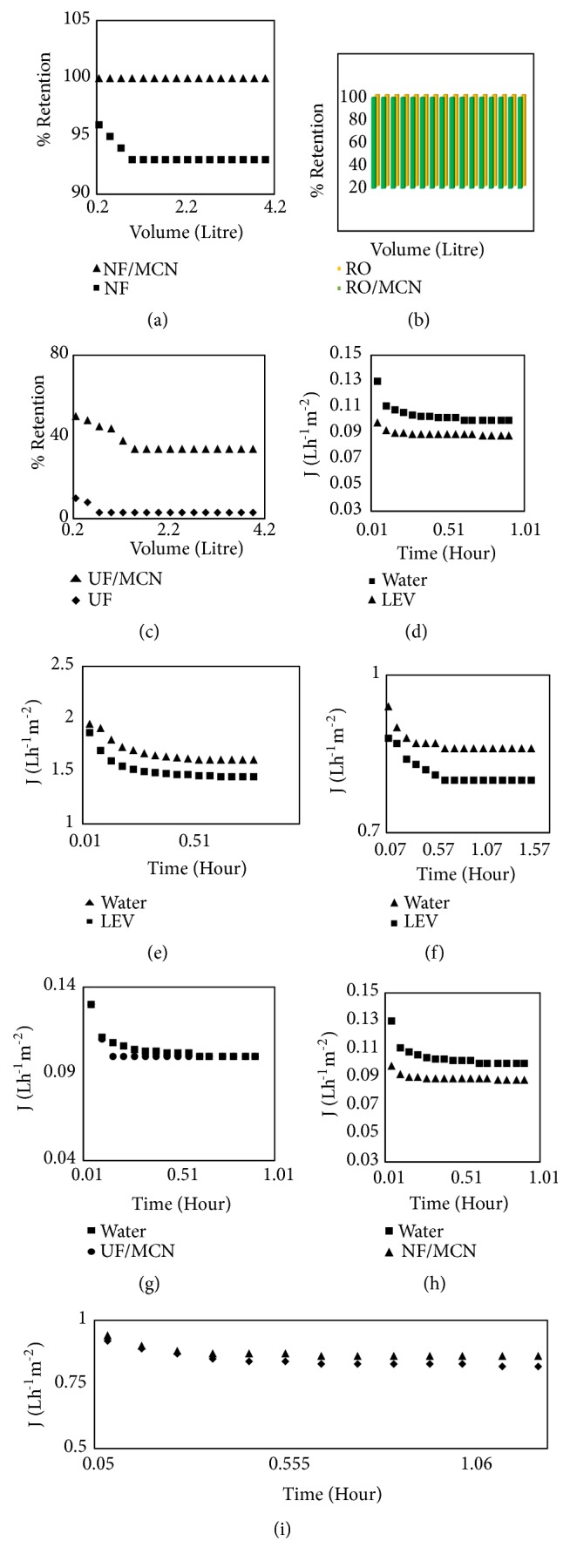
Effect of MCN on membrane parameters ((a)= percent retention by NF membrane and MCN/NF hybrid system, (b)= percent retention of LEV by R/O membrane and MCN/RO membrane in hybrid system, (c)=percent retention by UF membrane and MCN/UF hybrid system, (d)= effect of LEV on UF permeate flux, (e)= effect of LEV on NF permeate flux, (f)= effect of LEV on RO permeate flux, (g)= improved permeate flux of UF/MCN process, (h)= improved permeate flux of NF/MCN, and (i)= improved permeate flux in RO/ MCN hybrid system).

First the antibiotic solutions were allowed to pass through the three selected membranes. The percent retention of LEV by each membrane was calculated. Definitely high % R (almost 100%) was expected from NF and RO system as the MWCO (molecular weight cut-off) of these membranes are very low when compared to molecular weight of LEV (designated antibiotic). Around 90-96 % rejection was recorded with NF membrane system whereas 100% rejection was detected with RO membrane system (Figures 6(a) and 6(b)). The MWCO of the UF membrane was larger as compared to molecular weight of LEV. Consequently, lower percent retention was observed with naked UF membrane system (Figure 6(c)).

When the membranes were utilized in hybrid manner with MCN, the % R of RO membrane system was again 100%. The NF, % R went high up to 100%, while improvement in % R (from 5% with naked UF membrane to 40% with MCN/UF) still did not improve to 100%. However, UF membranes have high MWCO and allow most of the organic molecules to pass through it.

### 3.6. Effect of LEV on Permeate Flux of Membrane

The effect of LEV on permeate flux of three different types of membranes used are given in Figures 6(d), 6(e), and 6(f). It is clear from the figures that there was a drop in J at the preliminary phases for double distilled water through all the three nominated membranes. The drop in J is mainly due to the interaction/contact of hydrogen ions (H^+1^) and hydroxyl ions (OH^−1^) with membrane surface and inherent resistance of membrane. The value of electrical conductance for H^+1^ and OH^−1^ in double distilled water is 6.3 x 10^−5^ Sm^−1^ [32]. The values of J then touch a stable state and are never again influenced inside the experimental phase. The molecular mass (M) of LEV is lesser than MWCO of the UF membrane. LEV molecules are likely to pass spontaneously from UF membrane and the LEV concentration in permeate (C_p_) would be equivalent to that of the LEV concentration in bulk (C_b_) without addition of MCN in cross-breed/hybrid mode. Aside from low rejection of LEV molecules, drop in the value of J was also noted for LEV molecules.  Figure 6(g) displays the improved/enhanced J for MCN/UF. It is clearer from the figure that enhanced J was noted for the LEV molecules under study than naked UF membrane. Figures 6(h) and 6(i) display the improved/enhanced J of NF and RO membranes, respectively, as the molecular mass of LEV antibiotic is bigger than MWCO of the NF and RO membranes. These membranes practically retained 100% molecules of LEV antibiotic. As a result they have prominent and negative influence on J NF and RO membranes. When NF and RO membranes were operated in cross-breed/hybrid style with MCN in a specifically designed reactor, fairly improved permeate fluxes (J) were noted for both NF/MCN and RO/MCN cross-breed/hybrid manner (Figures 6(h) and 6(i)).

### 3.7. Back Wash Time of Membranes

After regular intervals of 60 minutes (experimental cycle) membranes were washed with distilled water. The time taken for backwashing of membranes with MCN synthesized from biomass precursors of pineapple was substantially less as compared with naked membranes because MCN was totally eliminated from the slurry by utilization of a magnet [31]. No darkening of the pipes and flow meters of the membrane systems was noted by utilization of MCN. In this manner MCN is valuable in membrane system and low-priced from an economic perspective because it decreases the backwashing time of membrane systems. Additionally, it has been set up from low-cost biomass precursors.

### 3.8. Adsorption Thermodynamics

For the determination of thermodynamics parameters the adsorption experiment was conducted at 298, 313, and 333K with LEV concentration of 20 mgL^−1^ and 0.05 g dosage of MCN. The Van't Hoff equation is utilized to calculate ΔH° and ΔS°:

(11) $$\begin{array}{c} \ln k=\frac{{\Delta H}^{o}}{R}-\frac{{\Delta S}^{o}}{RT} \end{array}$$

**Figure 7:**
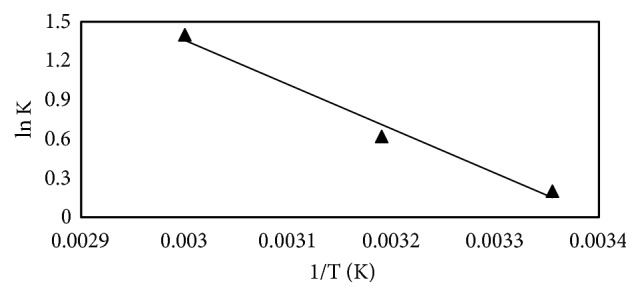
Van't Hoff plot of adsorption of LEV onto MCN.

In (11), ΔH° is standard enthalpy change, ΔS° is standard entropy change, T is the temperature in kelvin (K), R is universal gas constant (8.314 Jmol^−1^K^−1^), and k is distribution constant. The distribution constant (k) is calculated from quantity of LEV removed and equilibrium concentration of LEV(*k* = *C*_*e*_/*q*_*e*_).  Figure 7 displays the Van't Hoff plot and was achieved by plotting ln k versus I/T, with slope= -ΔH^o^/R and intercept = ΔS^o^/R.

The values of ΔG° (standard free energy change) were calculated using the following relation:

(12) $$\begin{array}{c} {\Delta G}^{o}={\Delta H}^{o}-{T\Delta S}^{o}. \end{array}$$

The different values calculated from the equation, -0.37, -1.81, and -3.73 kJ mol^−1^, correspond to 298, 303, and 333K, respectively. The negative values of ΔG° at various temperatures specify the spontaneous nature of the process and have a high affinity of LEV molecules for MCN.

## 4. Conclusion

In this study MCN was prepared from waste biomass and was tested for the removal of LEV from water. Pseudo-second-order and Langmuir models fitted the kinetics and equilibrium adsorption data excellently well amongst the used models. In hybrid membrane processes improved permeate flow and percent removal of LEV were observed. Blackening of membrane pipes, cake formation, and long backwashing time encountered with activated carbon were not observed in hybrid membrane system because MCN was effectively removed before entering into membrane was trapped through magnet. Therefore, the prepared MCN can be used as an alternative of activated carbon and can compete with most of the adsorbents in the field of surface chemistry for the removal of LEV from aqueous solution. From economical point of view, the use of MCN in membrane systems is inexpensive as compared with activated carbon because it decreases the backwashing time of membrane systems and does not causes blackening of membranes.

## Figures and Tables

**Table 1:** Characteristic properties of the Levofloxacin hemihydrate.

Structural formula	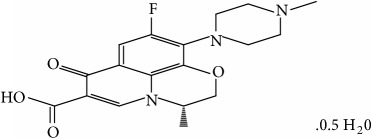
Chemical formula	C_18_H_20_FN_3_O_4_

IUPAC name	(-)-(S)-9-fluoro2,3dihydro-3-methyl-10-(4-methyl-1-piperazinyl)-7-oxo-7H-pyrido[1,2,3-de]-nzoxazine-6-carboxylic acid hemihydrate

Molecular mass	370.38 g/mol

Appearance	Yellowish white

Dissociation constant	6.24 (carboxylic acid moiety)

Solubility	Water soluble

**Table 2:** Characteristic properties of UF, NF, and RO membranes.

Membrane type	Parameters	Specification
*UF*	Material	Polyether sulfone
	Type	Capillary multi bores x 7
	Diameter bores ID	0.9 mm
	Diameter fiber OD	4.2mm
	MWCO	100KD
	Surface area	50 m^2^
	Maximum temperature	40°C
	Maximum pressure	7.5bar
	Membrane back wash pressure	0.5-1bar
	Operator pH range	3-10
	Back wash pH range	1-13
	Disinfection chemicals	
	Hypo chloride (NaOCl)	50-200 mgL^−1^
	Hydrogen peroxide (H_2_O_2_)	100-200 mgL^−1^

*NF* (DOW Film Tec 2.5 x 40)	Model	NF 270-2540
	Permeate Flow rate	3.2 m^3^/day
	Active surface area	28 ft^2^ (3.2 m^2^)
	Applied pressure	4.8 bar
	Stabilized salt rejection	> 97%

*RO* (DOW Film)	Model	TW 30-3812-40
	Membrane type	Thin film composite (filmtech)
	Permeate Flow rate	83.2 m^3^/day
	Active surface area	28 ft^2^ (3.2 m^2^)
	Maximum operating pressure	1.0 bar
	Stabilized salt rejection	99.5%
	pH range continuous operation	3-10
	pH range short term cleaning	1-12

**Table 3:** Kinetics parameters for adsorption of LEV onto MCN.

Concentration (mg/L)	Kinetic models	
20	*Pseudo 1st order*:	
	K_1_ (min^−1^)	0.056
	q_m_ (mg/g)	9.95
	R^2^	0.982

40	*Pseudo 1st order*:	
	K_1_ (min^−1^)	0.044
	q_m_ (mg/g)	12.53
	R^2^	0.993

20	*Pseudo 2nd order*:	
	K_2_ (gmg^−1^min^−1^)	0.026
	q_m_ (mg/g)	18.11
	R^2^	0.973

40	*Pseudo 2nd order*:	
	K_2_ (gmg^−1^min^−1^)	0.025
	q_m_ (mg/g)	16.25
	R^2^	0.995

20	*Intraparticle diffusion model*:	
	Kdiff	1.80
	C	3.70
	R^2^	0.943

40	*Intraparticle diffusion model*:	
	Kdiff	1.522
	C	1.60
	R^2^	0.943

**Table 4:** Isotherm parameters for adsorption of LEV onto MCN.

Isotherm	Parameter	Value
Langmuir	K_L_ (L/mg)	0.146
	q_m_ (mg/g)	20.75
	R^2^	0.984

Freundlich	K (mg/g) (L/mg)^1/n^	12.75
	1/n	1.1053
	R^2^	0.946

Tempkin	b	221.94
	*α*	0.76
	*β*	11.163
	R^2^	0.981

## Data Availability

The data used to support the findings of this study are available from the corresponding author upon request.
